# Supplementary material for: Pyruvate Dehydrogenase Kinase 1 inhibition mediated oxidative phosphorylation enhancement in cartilage promotes osteoarthritis progression
Source: BMC Musculoskelet Disord. 2023 Jul 20;24:597. doi: 10.1186/s12891-023-06585-6 (PMC10357736; doi:10.1186/s12891-023-06585-6)
Supplement: Supplementary file 1 — Additional file 1. [file 12891_2023_6585_MOESM1_ESM.zip › BP.Gsea.1653623667859/GOBP_DEVELOPMENT_OF_PRIMARY_FEMALE_SEXUAL_CHARACTERISTICS.html]

Details for gene set GOBP\_DEVELOPMENT\_OF\_PRIMARY\_FEMALE\_SEXUAL\_CHARACTERISTICS[GSEA]

|  || Dataset | 55457\_collapsed\_to\_symbols.cls.cls #OA\_versus\_normal.cls.cls #OA\_versus\_normal\_repos |
| Phenotype | cls.cls#OA\_versus\_normal\_repos |
| Upregulated in class | normal |
| GeneSet | GOBP\_DEVELOPMENT\_OF\_PRIMARY\_FEMALE\_SEXUAL\_CHARACTERISTICS |
| Enrichment Score (ES) | -0.42232224 |
| Normalized Enrichment Score (NES) | -1.8243047 |
| Nominal p-value | 0.0015243902 |
| FDR q-value | 0.08337712 |
| FWER p-Value | 0.95 |
Table: GSEA Results Summary

  

Fig 1: Enrichment plot: GOBP\_DEVELOPMENT\_OF\_PRIMARY\_FEMALE\_SEXUAL\_CHARACTERISTICS      
 Profile of the Running ES Score & Positions of GeneSet Members on the Rank Ordered List

  

| SYMBOL | TITLE | RANK IN GENE LIST | RANK METRIC SCORE | RUNNING ES | CORE ENRICHMENT || 1 | EIF2B2 | eukaryotic translation initiation factor 2B subunit beta [Source:HGNC Symbol;Acc:HGNC:3258] | 627 | 0.612 | -0.0268 | No |
| 2 | ESR1 | estrogen receptor 1 [Source:HGNC Symbol;Acc:HGNC:3467] | 984 | 0.522 | -0.0356 | No |
| 3 | ANG | angiogenin [Source:HGNC Symbol;Acc:HGNC:483] | 2009 | 0.360 | -0.1026 | No |
| 4 | KIT | "KIT proto-oncogene, receptor tyrosine kinase [Source:HGNC Symbol;Acc:HGNC:6342]" | 2117 | 0.348 | -0.0983 | No |
| 5 | SOD1 | superoxide dismutase 1 [Source:HGNC Symbol;Acc:HGNC:11179] | 2413 | 0.313 | -0.1100 | No |
| 6 | SFRP1 | secreted frizzled related protein 1 [Source:HGNC Symbol;Acc:HGNC:10776] | 2503 | 0.303 | -0.1060 | No |
| 7 | ACSBG1 | acyl-CoA synthetase bubblegum family member 1 [Source:HGNC Symbol;Acc:HGNC:29567] | 2520 | 0.301 | -0.0963 | No |
| 8 | GAS2 | growth arrest specific 2 [Source:HGNC Symbol;Acc:HGNC:4167] | 2866 | 0.264 | -0.1137 | No |
| 9 | PDGFRA | platelet derived growth factor receptor alpha [Source:HGNC Symbol;Acc:HGNC:8803] | 2943 | 0.256 | -0.1103 | No |
| 10 | CASP3 | caspase 3 [Source:HGNC Symbol;Acc:HGNC:1504] | 3202 | 0.230 | -0.1221 | No |
| 11 | SGPL1 | sphingosine-1-phosphate lyase 1 [Source:HGNC Symbol;Acc:HGNC:10817] | 3462 | 0.206 | -0.1349 | No |
| 12 | BRCA2 | BRCA2 DNA repair associated [Source:HGNC Symbol;Acc:HGNC:1101] | 3853 | 0.170 | -0.1591 | No |
| 13 | EIF2B4 | eukaryotic translation initiation factor 2B subunit delta [Source:HGNC Symbol;Acc:HGNC:3260] | 3878 | 0.167 | -0.1550 | No |
| 14 | ZFPM2 | "zinc finger protein, FOG family member 2 [Source:HGNC Symbol;Acc:HGNC:16700]" | 3958 | 0.160 | -0.1553 | No |
| 15 | CTNNA1 | catenin alpha 1 [Source:HGNC Symbol;Acc:HGNC:2509] | 4003 | 0.156 | -0.1531 | No |
| 16 | DMC1 | DNA meiotic recombinase 1 [Source:HGNC Symbol;Acc:HGNC:2927] | 4149 | 0.142 | -0.1593 | No |
| 17 | GDF9 | growth differentiation factor 9 [Source:HGNC Symbol;Acc:HGNC:4224] | 4318 | 0.127 | -0.1678 | No |
| 18 | ZP3 | zona pellucida glycoprotein 3 [Source:HGNC Symbol;Acc:HGNC:13189] | 4367 | 0.123 | -0.1671 | No |
| 19 | FSHB | follicle stimulating hormone subunit beta [Source:HGNC Symbol;Acc:HGNC:3964] | 4502 | 0.113 | -0.1735 | No |
| 20 | AMH | anti-Mullerian hormone [Source:HGNC Symbol;Acc:HGNC:464] | 5042 | 0.070 | -0.2130 | No |
| 21 | MSH4 | mutS homolog 4 [Source:HGNC Symbol;Acc:HGNC:7327] | 5055 | 0.069 | -0.2115 | No |
| 22 | SLIT3 | slit guidance ligand 3 [Source:HGNC Symbol;Acc:HGNC:11087] | 5134 | 0.061 | -0.2153 | No |
| 23 | FOXC1 | forkhead box C1 [Source:HGNC Symbol;Acc:HGNC:3800] | 5227 | 0.054 | -0.2205 | No |
| 24 | WT1 | WT1 transcription factor [Source:HGNC Symbol;Acc:HGNC:12796] | 5452 | 0.037 | -0.2367 | No |
| 25 | STAT5B | signal transducer and activator of transcription 5B [Source:HGNC Symbol;Acc:HGNC:11367] | 5554 | 0.028 | -0.2436 | No |
| 26 | LHFPL2 | LHFPL tetraspan subfamily member 2 [Source:HGNC Symbol;Acc:HGNC:6588] | 5575 | 0.026 | -0.2442 | No |
| 27 | BCL2 | BCL2 apoptosis regulator [Source:HGNC Symbol;Acc:HGNC:990] | 5609 | 0.023 | -0.2459 | No |
| 28 | NUPR1 | "nuclear protein 1, transcriptional regulator [Source:HGNC Symbol;Acc:HGNC:29990]" | 5616 | 0.023 | -0.2456 | No |
| 29 | GNRH1 | gonadotropin releasing hormone 1 [Source:HGNC Symbol;Acc:HGNC:4419] | 5621 | 0.022 | -0.2451 | No |
| 30 | FANCG | FA complementation group G [Source:HGNC Symbol;Acc:HGNC:3588] | 5675 | 0.017 | -0.2486 | No |
| 31 | LHCGR | luteinizing hormone/choriogonadotropin receptor [Source:HGNC Symbol;Acc:HGNC:6585] | 5679 | 0.017 | -0.2482 | No |
| 32 | INHA | inhibin subunit alpha [Source:HGNC Symbol;Acc:HGNC:6065] | 5701 | 0.016 | -0.2493 | No |
| 33 | FSHR | follicle stimulating hormone receptor [Source:HGNC Symbol;Acc:HGNC:3969] | 5757 | 0.011 | -0.2531 | No |
| 34 | ZFY | zinc finger protein Y-linked [Source:HGNC Symbol;Acc:HGNC:12870] | 5909 | 0.002 | -0.2649 | No |
| 35 | SPRR2A | small proline rich protein 2A [Source:HGNC Symbol;Acc:HGNC:11261] | 5922 | 0.001 | -0.2658 | No |
| 36 | BMPR1B | bone morphogenetic protein receptor type 1B [Source:HGNC Symbol;Acc:HGNC:1077] | 5959 | -0.002 | -0.2685 | No |
| 37 | FOXL2 | forkhead box L2 [Source:HGNC Symbol;Acc:HGNC:1092] | 6068 | -0.009 | -0.2766 | No |
| 38 | LFNG | LFNG O-fucosylpeptide 3-beta-N-acetylglucosaminyltransferase [Source:HGNC Symbol;Acc:HGNC:6560] | 6161 | -0.017 | -0.2832 | No |
| 39 | ATM | ATM serine/threonine kinase [Source:HGNC Symbol;Acc:HGNC:795] | 6179 | -0.019 | -0.2838 | No |
| 40 | CYP19A1 | cytochrome P450 family 19 subfamily A member 1 [Source:HGNC Symbol;Acc:HGNC:2594] | 6388 | -0.034 | -0.2988 | No |
| 41 | AFP | alpha fetoprotein [Source:HGNC Symbol;Acc:HGNC:317] | 6561 | -0.047 | -0.3106 | No |
| 42 | BCL2L1 | BCL2 like 1 [Source:HGNC Symbol;Acc:HGNC:992] | 6698 | -0.055 | -0.3192 | No |
| 43 | TNFAIP6 | TNF alpha induced protein 6 [Source:HGNC Symbol;Acc:HGNC:11898] | 6799 | -0.063 | -0.3247 | No |
| 44 | SRC | "SRC proto-oncogene, non-receptor tyrosine kinase [Source:HGNC Symbol;Acc:HGNC:11283]" | 6940 | -0.075 | -0.3329 | No |
| 45 | MMP2 | matrix metallopeptidase 2 [Source:HGNC Symbol;Acc:HGNC:7166] | 7037 | -0.082 | -0.3374 | No |
| 46 | IDH1 | isocitrate dehydrogenase (NADP(+)) 1 [Source:HGNC Symbol;Acc:HGNC:5382] | 7109 | -0.089 | -0.3398 | No |
| 47 | ARRB2 | arrestin beta 2 [Source:HGNC Symbol;Acc:HGNC:712] | 7255 | -0.100 | -0.3475 | No |
| 48 | SPO11 | SPO11 initiator of meiotic double stranded breaks [Source:HGNC Symbol;Acc:HGNC:11250] | 7436 | -0.114 | -0.3574 | No |
| 49 | BMP15 | bone morphogenetic protein 15 [Source:HGNC Symbol;Acc:HGNC:1068] | 7889 | -0.147 | -0.3874 | No |
| 50 | TAF4 | TATA-box binding protein associated factor 4 [Source:HGNC Symbol;Acc:HGNC:11537] | 7966 | -0.153 | -0.3878 | No |
| 51 | FZD4 | frizzled class receptor 4 [Source:HGNC Symbol;Acc:HGNC:4042] | 8159 | -0.168 | -0.3967 | No |
| 52 | EIF2B5 | eukaryotic translation initiation factor 2B subunit epsilon [Source:HGNC Symbol;Acc:HGNC:3261] | 8168 | -0.168 | -0.3912 | No |
| 53 | SLIT2 | slit guidance ligand 2 [Source:HGNC Symbol;Acc:HGNC:11086] | 8449 | -0.190 | -0.4062 | No |
| 54 | FST | follistatin [Source:HGNC Symbol;Acc:HGNC:3971] | 8511 | -0.194 | -0.4039 | No |
| 55 | NRIP1 | nuclear receptor interacting protein 1 [Source:HGNC Symbol;Acc:HGNC:8001] | 8524 | -0.195 | -0.3978 | No |
| 56 | LEP | leptin [Source:HGNC Symbol;Acc:HGNC:6553] | 8740 | -0.212 | -0.4069 | No |
| 57 | UBE3A | ubiquitin protein ligase E3A [Source:HGNC Symbol;Acc:HGNC:12496] | 8757 | -0.213 | -0.4004 | No |
| 58 | A2M | alpha-2-macroglobulin [Source:HGNC Symbol;Acc:HGNC:7] | 8876 | -0.223 | -0.4015 | No |
| 59 | SIRT1 | sirtuin 1 [Source:HGNC Symbol;Acc:HGNC:14929] | 8947 | -0.227 | -0.3987 | No |
| 60 | SMAD4 | SMAD family member 4 [Source:HGNC Symbol;Acc:HGNC:6770] | 9035 | -0.235 | -0.3970 | No |
| 61 | PGR | progesterone receptor [Source:HGNC Symbol;Acc:HGNC:8910] | 9048 | -0.235 | -0.3894 | No |
| 62 | UBB | ubiquitin B [Source:HGNC Symbol;Acc:HGNC:12463] | 9142 | -0.243 | -0.3879 | No |
| 63 | NUP107 | nucleoporin 107 [Source:HGNC Symbol;Acc:HGNC:29914] | 9274 | -0.253 | -0.3889 | No |
| 64 | FANCA | FA complementation group A [Source:HGNC Symbol;Acc:HGNC:3582] | 9293 | -0.255 | -0.3811 | No |
| 65 | BAX | "BCL2 associated X, apoptosis regulator [Source:HGNC Symbol;Acc:HGNC:959]" | 9323 | -0.257 | -0.3740 | No |
| 66 | PTX3 | pentraxin 3 [Source:HGNC Symbol;Acc:HGNC:9692] | 9943 | -0.316 | -0.4109 | Yes |
| 67 | NOS3 | nitric oxide synthase 3 [Source:HGNC Symbol;Acc:HGNC:7876] | 9980 | -0.319 | -0.4021 | Yes |
| 68 | NR2F2 | nuclear receptor subfamily 2 group F member 2 [Source:HGNC Symbol;Acc:HGNC:7976] | 10129 | -0.334 | -0.4016 | Yes |
| 69 | NR5A1 | nuclear receptor subfamily 5 group A member 1 [Source:HGNC Symbol;Acc:HGNC:7983] | 10308 | -0.351 | -0.4027 | Yes |
| 70 | CASP2 | caspase 2 [Source:HGNC Symbol;Acc:HGNC:1503] | 10506 | -0.374 | -0.4046 | Yes |
| 71 | RETN | resistin [Source:HGNC Symbol;Acc:HGNC:20389] | 10511 | -0.375 | -0.3913 | Yes |
| 72 | AMHR2 | anti-Mullerian hormone receptor type 2 [Source:HGNC Symbol;Acc:HGNC:465] | 10571 | -0.381 | -0.3821 | Yes |
| 73 | MMP19 | matrix metallopeptidase 19 [Source:HGNC Symbol;Acc:HGNC:7165] | 10652 | -0.388 | -0.3743 | Yes |
| 74 | PTPRN | protein tyrosine phosphatase receptor type N [Source:HGNC Symbol;Acc:HGNC:9676] | 10712 | -0.395 | -0.3646 | Yes |
| 75 | EREG | epiregulin [Source:HGNC Symbol;Acc:HGNC:3443] | 10778 | -0.403 | -0.3550 | Yes |
| 76 | HSPA5 | heat shock protein family A (Hsp70) member 5 [Source:HGNC Symbol;Acc:HGNC:5238] | 10820 | -0.407 | -0.3435 | Yes |
| 77 | PLEKHA1 | pleckstrin homology domain containing A1 [Source:HGNC Symbol;Acc:HGNC:14335] | 10899 | -0.416 | -0.3345 | Yes |
| 78 | INHBA | inhibin subunit beta A [Source:HGNC Symbol;Acc:HGNC:6066] | 11237 | -0.462 | -0.3440 | Yes |
| 79 | FOXO3 | forkhead box O3 [Source:HGNC Symbol;Acc:HGNC:3821] | 11305 | -0.472 | -0.3322 | Yes |
| 80 | KDR | kinase insert domain receptor [Source:HGNC Symbol;Acc:HGNC:6307] | 11454 | -0.496 | -0.3257 | Yes |
| 81 | COL9A3 | collagen type IX alpha 3 chain [Source:HGNC Symbol;Acc:HGNC:2219] | 11477 | -0.499 | -0.3093 | Yes |
| 82 | KITLG | KIT ligand [Source:HGNC Symbol;Acc:HGNC:6343] | 11516 | -0.503 | -0.2941 | Yes |
| 83 | DACH1 | dachshund family transcription factor 1 [Source:HGNC Symbol;Acc:HGNC:2663] | 11843 | -0.558 | -0.2993 | Yes |
| 84 | ARID5B | AT-rich interaction domain 5B [Source:HGNC Symbol;Acc:HGNC:17362] | 12187 | -0.640 | -0.3029 | Yes |
| 85 | MMP14 | matrix metallopeptidase 14 [Source:HGNC Symbol;Acc:HGNC:7160] | 12286 | -0.672 | -0.2862 | Yes |
| 86 | WNT4 | Wnt family member 4 [Source:HGNC Symbol;Acc:HGNC:12783] | 12331 | -0.686 | -0.2647 | Yes |
| 87 | ADAMTS1 | ADAM metallopeptidase with thrombospondin type 1 motif 1 [Source:HGNC Symbol;Acc:HGNC:217] | 12553 | -0.775 | -0.2539 | Yes |
| 88 | VEGFA | vascular endothelial growth factor A [Source:HGNC Symbol;Acc:HGNC:12680] | 12629 | -0.813 | -0.2303 | Yes |
| 89 | INHBB | inhibin subunit beta B [Source:HGNC Symbol;Acc:HGNC:6067] | 12680 | -0.851 | -0.2033 | Yes |
| 90 | TIPARP | TCDD inducible poly(ADP-ribose) polymerase [Source:HGNC Symbol;Acc:HGNC:23696] | 12789 | -0.950 | -0.1773 | Yes |
| 91 | ADCYAP1R1 | ADCYAP receptor type I [Source:HGNC Symbol;Acc:HGNC:242] | 12790 | -0.952 | -0.1428 | Yes |
| 92 | MYC | "MYC proto-oncogene, bHLH transcription factor [Source:HGNC Symbol;Acc:HGNC:7553]" | 12801 | -0.970 | -0.1084 | Yes |
| 93 | CEBPB | CCAAT enhancer binding protein beta [Source:HGNC Symbol;Acc:HGNC:1834] | 12804 | -0.973 | -0.0733 | Yes |
| 94 | INSR | insulin receptor [Source:HGNC Symbol;Acc:HGNC:6091] | 12856 | -1.071 | -0.0384 | Yes |
| 95 | PCYT1B | "phosphate cytidylyltransferase 1B, choline [Source:HGNC Symbol;Acc:HGNC:8755]" | 12877 | -1.151 | 0.0018 | Yes |
Table: GSEA details [plain text format]

  

Fig 2: GOBP\_DEVELOPMENT\_OF\_PRIMARY\_FEMALE\_SEXUAL\_CHARACTERISTICS      
 Blue-Pink O' Gram in the Space of the Analyzed GeneSet

  

Fig 3: GOBP\_DEVELOPMENT\_OF\_PRIMARY\_FEMALE\_SEXUAL\_CHARACTERISTICS: Random ES distribution      
 Gene set null distribution of ES for **GOBP\_DEVELOPMENT\_OF\_PRIMARY\_FEMALE\_SEXUAL\_CHARACTERISTICS**
